# Evaluation of the Adsorption Efficacy of Bentonite on Aflatoxin M_1_ Levels in Contaminated Milk

**DOI:** 10.3390/toxins15020107

**Published:** 2023-01-26

**Authors:** Gamal M. Hamad, Hussein S. Abo El-Makarem, Marwa G. Allam, Osama S. El Okle, Marwa I. El-Toukhy, Taha Mehany, Yasser El-Halmouch, Mukhtar M. F. Abushaala, Mohamed S. Saad, Sameh A. Korma, Salam A. Ibrahim, Elsayed E. Hafez, Amr Amer, Eman Ali

**Affiliations:** 1 Department of Food Technology, Arid Lands Cultivation Research Institute, City of Scientific Research and Technological Applications, Alexandria 21934, Egypt; 2 Department of Food Hygiene and Control, Faculty of Veterinary Medicine, Alexandria University, Alexandria 22758, Egypt; 3 Department of Food Science, Faculty of Agriculture (Saba Basha), Alexandria University, Alexandria 21531, Egypt; 4 Department of Forensic Medicine and Toxicology, Faculty of Veterinary Medicine, Alexandria University, Alexandria 22758, Egypt; 5 Food Hygiene & Control Department, Faculty of Veterinary Medicine, Mansoura University, Mansoura 35516, Egypt; 6 Department of Chemistry, University of La Rioja, 26006 Logroño, Spain; 7 Department of Botany and Microbiology, Faculty of Science, Kafrelsheikh University, Kafr Elsheikh 33511, Egypt; 8 Department of Food Hygiene, Faculty of Veterinary Medicine, Azzaytuna University, Tarhuna 22131, Libya; 9 Department of Food Science, Faculty of Agriculture, Zagazig University, Zagazig 44519, Egypt; 10 School of Food Science and Engineering, South China University of Technology, Guangzhou 510641, China; 11 Food Microbiology and Biotechnology Laboratory, Food and Nutritional Sciences Program, College of Agriculture and Environmental Sciences, North Carolina A & T State University, Greensboro, NC 27411-1064, USA; 12 Plant Protection and Biomolecular Diagnosis, Arid Lands Cultivation Research Institute, City of Scientific Research and Technological Applications, Alexandria 21934, Egypt; 13 Department of Food Hygiene, Faculty of Veterinary Medicine, Damanhour University, Damanhour 22511, Egypt

**Keywords:** mycotoxins, AFM_1_, milk quality, milk safety, cytotoxicity, adsorbent, bentonite

## Abstract

The existence of aflatoxin M_1_ (AFM_1_) in raw milk results in economic losses and public health risks. This research aims to examine the capability of bentonite to adsorb and/or eliminate AFM_1_ from various raw milk types. In addition, the effects of numerous bentonites (HAFR 1, 2, 3 and 4) on the nutritional characteristics of the milk were studied. Our findings revealed that goat milk had the highest value of AFM_1_ (490.30 ng/L) in comparison to other milks. AFM_1_ adsorption was influenced by applying bentonite (0.5 and 1 g) in a concentration-dependent manner for different time intervals (from 0 to 12 h). The percentage of AFM_1_ reached the maximum adsorption level after 12 h to 100, 98.5 and 98% for bentonites HAFR 3, 1 and 2, respectively. HAFR 3 (1 g bentonite) presented higher adsorption efficiency than other bentonites used in the phosphate buffer saline (PBS) and milk. Residual levels of AFM_1_ reached their lowest values of 0 and 1.5 ng/L while using HAFR 3 in PBS and milk, respectively. With regard to the influence of bentonite on the nutritional characteristics of milk, there was an increase in fat, protein and solid non-fat ratio while using HAFR 3 and 4, yet decreased lactose in comparison with the control. Scanning Electron Microscopy and Fourier Transform-Infrared Spectroscopy both identified bentonites as superior AFM_1_ binders. The results demonstrated that bentonite, particularly HAFR 3, was the most effective adsorbent and could thus be a promising candidate for the decontamination of AFM_1_ in milk.

## 1. Introduction

According to the World Health Organization, one of the most well-known and extensively studied categories of mycotoxins, aflatoxins (AFs), are present as pollutants in food products throughout the world [1,2]. Numerous fungal species, including *Aspergillus flavus*, *Aspergillus parasiticus* and *Aspergillus nomius,* which grow in many food crops under a variety of climatic circumstances, create AFs as poisonous byproducts of the secondary metabolism of these fungi [3,4]. *A. flavus* and *A. parasiticus* produce aflatoxins of four various types: G_1_, G_2_, B_1_ and B_2_. The most potent naturally occurring genotoxic and carcinogenic toxin is called aflatoxin B_1_ (AFB_1_). Mycotoxins are commonly found in various cereal crops and other food stuffs, and are processed in the liver [5]. The primary oxidized metabolite of AFB_1_ is AFM_1_, which is present in milk (more specifically, in the water and cream portions) and other dairy foods where it may be persuaded by feed carry-over contamination [6]. The most concerning challenge related to aflatoxins is that they cannot be destroyed by traditional food processing methods including fermentation, refrigeration, heating, freezing or pasteurization [7]. Thus, AFM_1_ is a potential health risk, especially for children, when it is present in raw milk and some dairy products [8]. The most frequent side effects of AFM_1_ exposure are teratogenicity, hepatotoxicity and immunotoxicity caused by eating contaminated food and feed [9,10]. In order to limit exposure to AFM_1_, its maximum allowable levels in milk (50 ng/kg) have been determined by the European Union https://www.legislation.gov.uk/eur/2006/1881 (accessed on 14 September 2022) and many other nations [11].

Bentonites, such as montmorillonite, are heterogeneous deposits made of colloidal and elastic clays formed by volcanic tephra [12]. Bentonites possess a high montmorillonite content and work well as adsorbents for a variety of pollutants [13]. These substances are plentiful in nature, have selective adsorption, are inexpensive, and, most importantly, have low toxicity [14].

In order to reduce the amount of AFs in food and/or feed, several approaches such as physical techniques, biological inactivation and fermentation have been developed and implemented [15,16]. However, none of these methods has yet been proven to be 100 percent effective, secure or practical from an economic standpoint. Another approach is to use non-toxic ingredients, such as bentonites, to bind aflatoxins in feed and food and while maintaining the nutritional value, functionality and organoleptic quality of the food [17,18,19].

Mineral clay-based adsorbents, i.e., activated carbon (charcoal), zeolite, saponite-rich bentonite and HSCA offer an effective decontamination approach and are able to bind AFs, consequently decreasing the absorption of AFB_1_ in the gastrointestinal tract and its carry-over as AFM_1_ in dairy [20]. Montmorillonite clay can be effectively modified with plant extracts for the decontamination of T-2 toxin [21]. Similarly, clay adsorbents have been repeatedly proposed as adsorbents for treatment purposes, but natural clays are hydrophilic and can be inefficient for catching hydrophobic pharmaceuticals [22].

Moreover, some researchers have suggested using an adsorbent such as soil bentonite to reduce or detoxify aflatoxin from contaminated milk. It has been demonstrated that these elements are effective at lowering AF levels [23]. Most recently, we investigated the adsorption efficacy of calcium and sodium bentonites for ochratoxin A (OTA) in several cheese samples and concluded that calcium and sodium bentonites could be applied as an innovative food-grade adsorbent for OTA. Moreover, novel enriched feta cheese with calcium bentonite displayed the highly superior organoleptic properties [19].

The viability of any aflatoxin decontamination method will depend on how well the method works and how expensive it is to use. However, there are differences in the effectiveness of using physical, biological and chemical technologies for decontamination alone or in combination. Physical methods are less practical than biological ones since the bacteria have fewer degrading processes and are more stable in the gastrointestinal (GI) tract at various pH values [24]. As a result, the best adsorbents for aflatoxin sequestration are chemically inert bentonite clays. These clays have outstanding physical and chemical characteristics and are frequently used in the food industry. These characteristics include surface specificity, enlargement, adsorption, cation exchange, low cost, high safety and rheological and colloidal features [25].

Testing the adsorption affinity of bentonite against AFM_1_ is known as one of the commonly approaches for lowering AFM_1_ in raw milk. Therefore, the aim of this research was to test the ability of various bentonite types as an effective binding/adsorbing agent for AFM_1_ found in contaminated milk.

## 2. Results and Discussion

### 2.1. Spectroscopic and Microscopic Characterization of Bentonites

**Figure 1:**
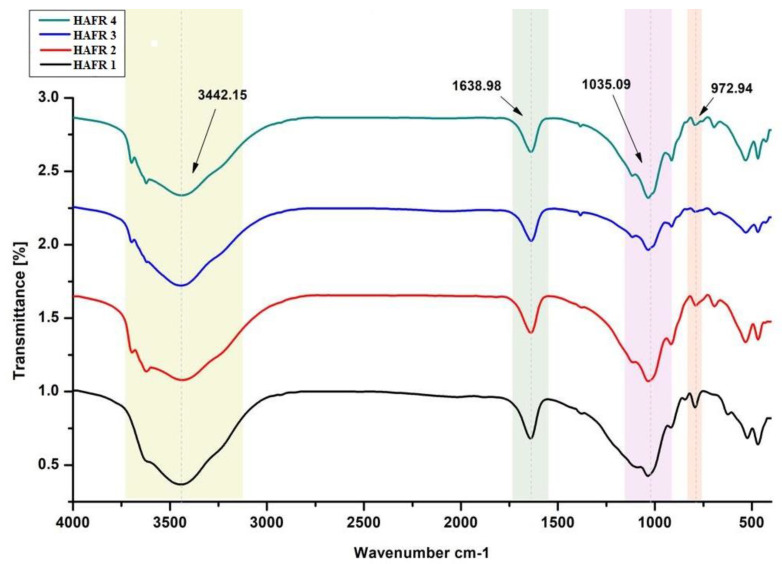
Fourier Transform Infrared (FTIR) of different types of bentonites.

In the present research, FTIR has been utilized to detect the chemical aspects of resulted peak and settle efficacious enclosure of all the tested components of the four examined bentonites across the spectrum ranging between 972–3442 cm^−1^. In Figure 1, infrared absorption spectra show the typical peaks of C=C, C=N and C-H in ring structure at 972 cm^−1^, C-O of carbohydrate at 1035 cm^−1^ and acetylated amide at 1753 cm^−1^. Whereas, the typical peaks are stated as C-O stretching of amide I at 1638 cm^−1^, OH of carbohydrates, proteins and polyphenols at 3442 cm^−1^. Bentonites showed abroad band at 3442.15 cm^−1^ because of -OH- stretching band for inter-layer adsorbed water (water current in the mineral bentonites). This implies the opportunity of the hydroxyl linkage among octahedral and tetrahedral layers. From the current findings, we noticed that a very sharp band detected at 1638.98 cm^−1^ is due to the asymmetric -OH- stretch (deformation mode) of water and a structural part of the mineral, while bands at 1035.09 cm^−1^ and 972.94 cm^−1^ were formed by the stretching mode of Si-O (out-of-plane) and Si-O stretching (in-plane) vibration for layered silicate.

**Figure 2:**
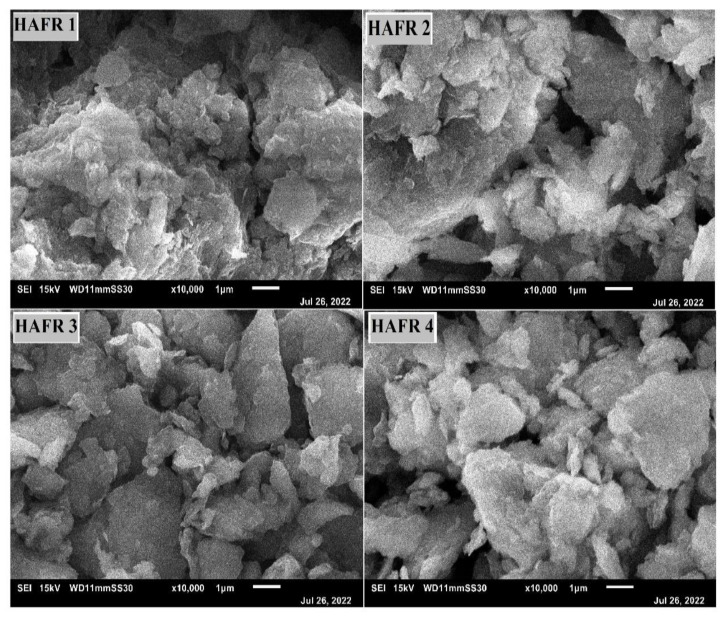
Scanning Electron Microscopy (SEM) of different bentonites (HAFR 1, HAFR 2, HAFR 3 and HAFR 4).

Scanning electron microscopy (SEM) and transmission electron microscopy (TEM) were applied to investigate the surface morphology of bentonites. The four bentonites were identified by scanning electron microscopy as being similar in appearance and having sizes, which ranged between 100 and 200 nm. The obtained SEM results presented in (Figure 2) revealed a significantly rougher surface, obstructed with few cracks. These types of surfaces indicate that the fracture toughness of the four examined bentonites could be improved. Images taken with a scanning electron microscope reveal shape irregularities and the dispersion of clay minerals, which may primarily be silica and alumina.

**Figure 3:**
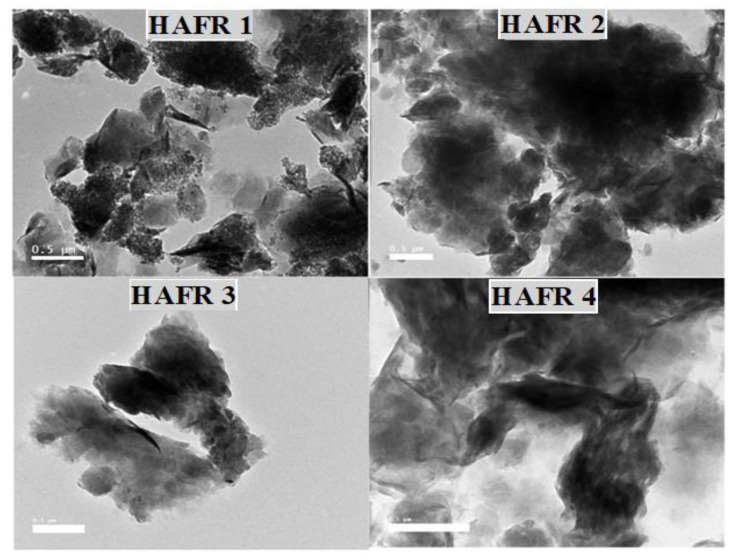
Transmission Electron Microscope (TEM) of different bentonites (HAFR 1, HAFR 2, HAFR 3 and HAFR 4).

Concerning TEM analysis (Figure 3), it was observed that the diameters of the bentonites range from 100 to 200 nm. The particles’ surface looks like graphite particles with rough borders. The particles are aggregated due to their chemical structure, which harbors many elements, for instance NA, Mg, Al, Si, Cl, K, Ca and Ti, as shown in Table 1. The ratio of the elements Si, Al and Na was high. Meanwhile, Cl was absent in HAFR 1 and 3, and Ti was absent in HAFR 1 (Table 1). This consequence shows the existence of alumina and silica as major ingredients along with traces of Fe, Mg, Na, K, Ti and Ca. The current findings are in line with those reported by various recent studies [26,27,28,29].

The massive changes in the internal surface area between the different types of bentonites (HAFR 3 and 1) are considered the effective parameter that provides abundant sites for attachment. These sites are capable of binding with the AFM_1_ as a protein and work to diminish it from the treated samples under this investigation. According to Pеtrovi et al. [30], bentonite’s adsorption properties depend mainly on its chemical structures and mineralogical structure, along with its textural and morphological features. Additionally, these minerals have numerous applications in various industrial processes due to their properties, which include surface area, surface charges, the type of exchangeable cations, hydroxyl groups on the edges, charge density, Lewis and Bronsted acidity and silanol groups of crystalline defects or broken surfaces [31]. Thus, these studies confirm our finding that the adsorption ability of bentonites towards AFM1 is dependent mainly on the bentonite’s properties; thus, in the current study, only two bentonites among the four examined showed high activity in aflatoxin removal compared with the other two bentonites. Moreover, the existence of calcium in the four examined bentonites was very low; therefore, it is recommended that these bentonites be used for a different purpose, as Calcium bentonite is less commonly used, as reported by studies on the impact of different bentonite complexes on the removal of aromatic compounds, which is an important indicator that the composition of the used bentonite could be a marker for its removal activity.

Moreover, recent research concluded that bentonites offer great potential to control aflatoxins and improve food and dairy safety and quality [32,33]. Conversely, the AFM_1_ distribution in milk has non-homogenous aspects, and a significant amount is bound to the milk protein (casein) [34]; this highlight makes the mechanism of adsorption more complex by various bentonite HAFR types. Therefore, the selection of the HAFR type according to its properties provides great potential for adsorbing AFM_1_ in milk.

### 2.2. Concentration and Frequency of Detection of Aflatoxins M_1_ in Raw Milk Samples Using HPLC

Several studies have shown that most raw milk may be contaminated with some types of aflatoxins, especially aflatoxin M_1_ [11,35]. Many physical, chemical and biological procedures have been applied to get rid of these toxins and their effects, but no methods have succeeded at a satisfactory rate. Therefore, in this study, we tried to use a method to get rid of aflatoxin in milk using four types of Egyptian bentonites. Data presented in Table 2 illustrate that the percentage of AFM_1_ found in cow, camel, sheep and goat milk samples were 88, 80, 86.7 and 93.3%, with mean values of 54.7, 87.5, 168.4 and 237.1 ng/L, respectively. European Commission No 1881/2006 established that the highest residue amount of AFM_1_ must not be more than 50 ng/L; though 54, 70, 73.3 and 86.7%, respectively, of the examined cow, camel, sheep and goat milk samples were above the level recommended by European Commission.

The greatest amount of contamination was observed in goat milk (490.3 ng/L), with a range of 5.60–490.30 ng/L and a mean value of 237.08 ng/L. These findings concurred with the findings acquired by de Matos et al. [35], who reported that 100% of examined Brazilian goat milk was contaminated with AFM_1_, with a mean value of 21.90 ± 10.28 ng/L. In addition, Omar [11] discovered that sheep milk had the greatest levels of AFM_1_ contamination, and recorded a value of 137.18 ng/kg, with a mean value of 70.25 ± 14.85 ng/kg.

The present study’s results are highly congruent with those reported by Elzupir and Elhussein [36], who observed that 95.45% of cow milk produced in Khartoum city, Sudan was contaminated with AFM_1_. Similarly, the lower incidence of aflatoxin in camel milk was found by Hussain et al. [37], who investigated AFM_1_ contamination in milk from five dairy species in Pakistan and discovered that camel milk was free from AFM_1_. They also discovered that 37.5, 20 and 16.7% of the examined cow, goat and sheep milk samples were contaminated with AFM_1_.

#### 2.2.1. Adsorption Capability of AFM_1_ in PBS Solution by Various Bentonite HAFR Types

For aflatoxins M_1_ adsorption capability via different bentonite concentrations, Table 3 displays AFM_1_ adsorption by different ratios of bentonite (0.5 and 1 g) in PBS. AFM_1_ adsorption was influenced by applying bentonite (0.5 and 1 g) in a concentration-dependent manner over different durations of time, i.e., 0, 0.5, 1, 2, 3, 6 and 12 h. Residual levels of AFM_1_ reached their lowest value of 0 ng/L in HAFR 3 (1 g bentonite), followed by 1.5 ng/L with HAFR 1 (1 g bentonite) and 2 ng/L with HAFR 2 (1 g bentonite). In details and with HAFR 3, they were 100 ng/L at 0 h > 60 ng/L at 0.5 h > 38 ng/L at 1 h >18 ng/L at 2 h > 5 ng/L at 3 h > 1 ng/L at 6 h >0 ng/L at 12 h. The highest adsorption percentage of AFM_1_ reached the maximum level after 12 h to 100, 98.5 and 98% while using bentonites HAFR 3, 1 and 2, respectively (Table 4).

Adsorbent concentration has an impact on adsorption mechanisms and can alter the dynamic sites available for mycotoxin adsorption. The maximum AFM_1_ adsorption capacity in this investigation was attained by employing a high concentration of 1 g of bentonite (HAFR 3, then HAFR 1). This indicates that when more bentonite was added to the milk, the amount of AFM_1_ that was adsorbed increased. These data are in agreement with the findings of Applebaum and Marth [38], who reported that 0.4 g of bentonite per 20 mL of naturally contaminated raw whole milk (at 25 °C for 1 h) was sufficient to accomplish the maximal removal of AFM_1_ (89%) by bentonite. Moreover, according to Jaynes et al. [39], bentonite can swell up to six times its original size when activated by water, which makes it extremely absorbent and useful for drawing out pollutants.

Time response is a physicochemical mechanism of adsorption that involves moving weights from liquid to solid surfaces [40]. AFM_1_ was quickly sequestered by bentonite in this investigation, especially at higher doses, with the adsorption percentage peak accumulating after 12 h, according to the adsorption versus time plots. Adsorption behavior revealed that AFM_1_ was rapidly absorbed in the first 0.5 h and then grew progressively until reaching its peak after 12 h. These findings might support the idea that there are enough vacant adsorption sites open to AFM_1_ molecules throughout the earliest stages of their reactivity with bentonite up until total adsorbent surface saturation. The subsequent decrease in AFM_1_ is brought on by the repulsive forces between molecules linked to active regions on the surface of the bentonite [41].

Results presented in Table 5 demonstrate the AFM_1_ adsorption capability obtained by (1 g bentonite) HAFR 1 and 3 in milk, where no adsorption was observed for untreated milk and all controls and positive control continually measured 100 ng/L AFM_1_ over different time intervals. After using HAFR 3, the concentration of AFM1 reached the lowest level after 12 h at 1.5 ng/L, followed by HAFR 1, which reached 5 ng/L after 12 h. Collectively, the adsorption percentage for HAFR 3 (98.5%) was relatively higher than that obtained for HAFR 1 (95%), as displayed in Table 6. Our results agreed with Abdel-Wahhab and Kholif [42], who claimed that supplementing early lactating goats with sodium bentonite 1% can significantly reduce the amount of AFM_1_ in the milk. Furthermore, bentonite, one of the well-known absorbents, can lower the levels of aflatoxin in milk owing to its wide variety and stable way of binding to AFM_1_ [43]. According to Montaseri et al. [44], milk from cows and goats fed a diet treated with bentonite had a considerably lower AFM_1_ content.

### 2.3. Effect of Bentonites on Qualitative Characteristics of the Milk Samples

Regarding the qualitative aspects of the milk samples, Table 7 illustrates the impact of 1 g bentonites (HAFR 1, 2, 3 and 4) on the chemical properties of milk. We can notice that there is an increase in fat, protein and SNF content while using HAFR 3 and 4, but a decreasing trend in lactose was observed compared with negative control. The milk composition of treated milk with HAFR 3 and HAFR 4 was significantly increased in fat and SNF compared with the control, while protein and lactose were reduced according to different bentonite concentrations. The action of bentonite on milk composition may be due to the alterations that occur in milk composition after the treatment with bentonites. Our findings agreed with Awasthi [45], who reported that bentonite may lower milk proteins. Hence, Abdel-Wahhab and Kholif [42] demonstrated that supplementing early lactation goats with 1% bentonite had no effect on the milk’s composition. Another study postulated that bentonite has a low influence on the nutritional characteristics of milk [46].

### 2.4. Cytotoxicity Evaluation of Bentonites

The estimation of cytotoxicity of bentonite and IC_50_ (µg/mL) is presented in Table 8. The type of bentonite and its concentration had an impact on the variation in inhibition. At a maximal concentration of 500 µg/mL, bentonite was more cytotoxic, with 100% inhibition and zero viability of the cell in all four examined types. Most cells are still alive at the smallest concentration (0.97 µg/mL) of bentonite, with a very low inhibitory percentage (2 and 6%) for HAFR 1 (98%) and HAFR 3 (94%), respectively. The required level of four types of bentonites for 50% inhibition of cell (IC_50_) was 57.1, 16.5, 11.35 and 6.92 for HAFR 1, 2, 4 and 3, respectively. This means that HAFR 1 is the most toxic and HAFR 3 is the least cytotoxic. Furthermore, the maximum and minimum toxic constituents are intended to be detected by the cytotoxic evaluation. To select the initial bentonite dosage for cytotoxic activity, the neutral red assay can estimate IC_50_ [19]. In this study, bentonite was exceedingly hazardous at the highest dosage of 500 µg/mL, while being comparatively non-toxic at the lowest concentration of 0.97 µg/mL. At a dosage of 1.95 µg/mL, there was minimal cytotoxicity. This result agrees with that of Zhang et al. [47], who discovered that the bentonite cytotoxicity on human B lymphocytes increases with an increase in bentonite dose and acquaintance time. The ability of HAFR 1 and 3 to be employed as non-toxic candidates is improved by their low cytotoxicity. The variation of cytotoxicity among the four examined bentonites may be due to the differences in their chemical structures, particle sizes and surface etiologies. The surface characteristics, morphology, and chemical structures of the adsorbent all affect how toxicant induction occurs in cell lines [48]. For studying the chemical, surface and morphological characterization of the four examined bentonites, XRD, FTIR, SEM and TEM will be discussed in the following sections.

### 2.5. X-ray Diffraction of Different Types of Bentonites

**Figure 4:**
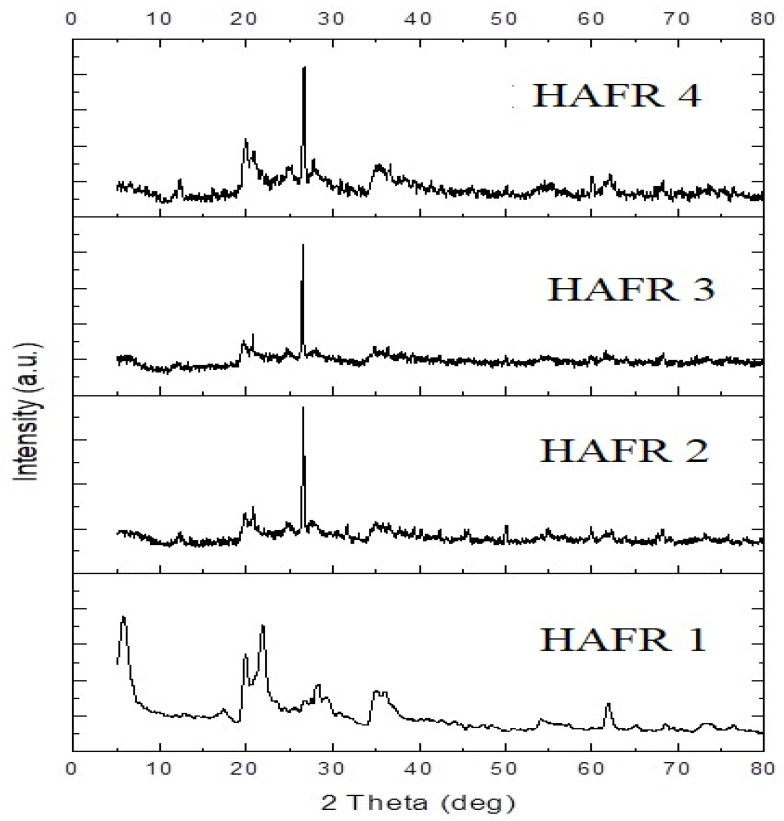
X-Ray diffraction of different types of bentonites (HAFR 1, HAFR 2, HAFR 3 and HAFR 4).

Figure 4 illustrates the characteristics of crystalline peaks (2 theta) of the four tested bentonites assessed by XRD powder analysis, and their reflections were recorded as follows: 20.2 = (101), 28.6 = (111), 40.23 = (200) and 60.43 = (220). However, the second beak was moved to the left in sample number 1, and was found at 23.2° instead of 28.6°, as shown in the other three bentonites. Samples’ basal reflections at angles (i.e., 20.20°, 28.6°, 40.23° and 60.43°) were observed, which are associated with the presence of impurities, such as aflatoxin, in samples. As shown in Figure 4, bentonite nanoparticles have numerous phases of low quartz and anortithe. This deflection peak has an index miller as follows: 101, 111, 200 and 220. Moreover, it displays that both samples have the phase of low quartz with trigonal structures and anortithe with triklin structures [49]. Therefore, the residual spreading peaks may be because of the crystalline structure and the interaction of the utilized *C. colocynthis* components [50].

## 3. Conclusions

The adsorption efficiency of various bentonites with aflatoxin M_1_ in milk was highlighted. In addition, the X-Ray, spectroscopic and microscopic characteristics of bentonites were assessed. Among the tested milk samples, goat milk was heavily contaminated with AFM_1_. HAFR 3 (1 g bentonite) had higher adsorption efficiency than other bentonite kinds used in phosphate buffer saline and milk, followed by HAFR 1. With regard to the impact of bentonite on the nutritional characteristics of milk, HAFR 3 and 4 caused moderate changes in fat, protein and SNF. On the other hand, lactose concentration decreased in comparison with the control. Due to their structure and their pH features, HAFR 3 and HAFR 1 demonstrated a high capability to adsorb high amounts of aflatoxin M_1_. Therefore, the current study confirms that bentonite’s adsorption properties depend on its chemical and mineralogical composition. Based on our results, HAFR 3 and HAFR 1 could be used as promising potential additives to decrease the risks of aflatoxin contamination in raw milk or other fresh food products.

## 4. Materials and Methods

### 4.1. Chemicals and Reagents

AFM_1_ standard solutions (50 µg mL^−1^ in acetonitrile) were obtained from (Sigma-Aldrich, Dorset, UK). HPLC-grade acetonitrile and methyl alcohol were purchased from (Sigma-Aldrich, Steinheim, Germany). Raw milk samples were acquired from local farms in various regions of Alexandria Governorate, Egypt. The preliminary milk sample was prepared in aliquots of 50 mL and then stored at −20 °C for further analysis.

### 4.2. Sampling

A total of 100 samples of raw milk from i.e., cow (n = 50), camel (n = 20), sheep (n = 15) and goat (n = 15) were randomly collected within 5 months, obtained from November 2021 to March 2022 from different regions of Alexandria City-Egypt for AFM1 analysis. All samples were transported to the laboratory in an icebox at 2–4 °C. The milk samples were sterilized prior to AFM1 contamination at 140 °C for 2 s. An measurement of 30 mL milk was placed in a dark flask and kept refrigerated until analysis.

#### Sample Preparation

For milk preparation, the samples were centrifuged (ThermoFisher Scientific Co., Cairo, Egypt) at 3500× *g* for 10 min at 10 °C. The upper phase was removed by a Pasteur pipette for further testing [51].

### 4.3. Cleanup/Purification and HPLC Conditions

The extraction of AFM_1_ from milk was accomplished according to the procedure of Iqbal et al. [52], with slight modifications, and the test kit of immunoaffinity columns was also utilized. The samples were first prepared (see Section 2.2.1). The extracts were then filtered with No. 5 Whatman filter paper, and approximately 50 mL of the sample was passed through an AflaTest immunoaffinity column at a rate ranging between 1–3 mL/min, then washed with 10 mL of deionized water (Milli Q Siemens, Ultra Clear, UV UF TM, Munich, Germany). Further, the bound AFM_1_ was eluted with acetonitrile reagent (1.5–3.0 mL). Finally, the residue was evaporated (40 °C) with a nitrogen stream. The prepared samples were placed in a dark place for (15 min) at RT. Then, 200 μL of acetonitrile was added to the vials. A 20 μL portion of the solution was subjected to LC analysis. The HPLC system (Agilent 1100 series system, Agilent Technologies, Hewlett-Packard, Waldbronn, Germany) included a model G1311A gradient delivery pump with G1321A fluorescence detector set at ƛʎ λex 360 and λem 430 nm. A Zorbax Eclipse XDB-C8 analytical column (4.6 mm × 150 mm, 5 µm; Agilent Technologies, Santa Clara, CA, USA). The mobile phase was isocratic water: acetonitrile: MeOH (68:24:8, *v*/*v*/*v*), and all separations were conducted at a flow rate of 1.0 mL/min [53]. The calibration curve on HPLC was carried out using several concentrations of AFM_1_. The detection level limit was 1.4006, hence, the limit of quantification was 1.8116. On the other hand, the AFM_1_ linear range ranged from 0.1–100 ng/mL.

### 4.4. Adsorption of AFM_1_ in Phosphate Buffer Saline (PBS) by Different Types of Bentonite Samples

AFM_1_ was used as a contaminant solution. The PBS of 100 ng/L AFM_1_ was exposed to numerous amounts of bentonite (obtained from Egypt’s New Borg El Arab bentonite group) equal to 0.5 and 1 g for several durations i.e., 0, 0.5, 1, 2, 3, 6 and 12 h. Different quantities of bentonite (HAFR 1, HAFR 2, HAFR 3 and HAFR 4) were added to PBS (15 mL) of AFM_1_ (100 ng/L). The adsorption assessments were performed with constant agitation at RT [19,51]. The adsorbent was separated using a centrifuge, and later, the supernatant was utilized for HPLC analysis, as designated in Section 2.3. The AFM_1_ removal efficiency (RE) was calculated by the following equation (Equation (1)):

RE (%) = (C0 − C1)/C0 × 100(1)

where RE (%) signifies of AFM_1_ removal ratio, and C0 and C1 are the concentration of AFM_1_ before and after adsorption, correspondingly.

### 4.5. Adsorption of AFM_1_ in Milk Samples by Bentonite (HAFR 1 and HAFR 3)

The bentonite (HAFR 1 and HAFR 3) was utilized to determine of AFM_1_ adsorption level in milk samples contaminated with AFM_1_ (100 ng/L). The amounts of 1 g of bentonite (HAFR 1 and HAFR 3) were added to 30 mL of contaminated milk. The dispersions were shaken vigorously for 12 h. The samples were centrifuged, and the upper part of the milk samples was investigated. The AFM_1_ quantities were examined by HPLC (HPLC–FLD) as clarified in Section 2.3. [19,54].

### 4.6. Qualitative Properties of the Tested Milk

Protein, lactose, fat and solid not fat (SNF) ratio in the milk samples was measured using Milk Scan (Broker Optik GMBH, MIRA model, Lenggries, Germany). To contaminate the milk, 100 ng/L of AFM_1_ was added. Moreover, the adsorbent equal to 1 g was considered to evaluate the adsorption rate for different types of bentonites compared to the positive and negative control [54].

### 4.7. Cytotoxicity Assessment of Bentonites

Cell viability was examined using peripheral blood mononuclear cells (PBMCs) maintained in the RPMI medium. The PBMCs approach utilizes cells that are isolated from multiple individuals, and could provide a high assessment of the cytotoxicity of drugs in vitro. Moreover, PBMCs cell types provide a principal reflection into how immune cell from several donors respond to the candidate constituents in development. Blank wells (150 µL PBS), control wells (150 µL PBMCs) and tested wells (150 µL PBMCs) were placed on a microtiter plate. Bentonite at several ratios was added to test wells and incubated (New Brunswick™ Galaxy^®^ 170 R CO2 Incubator Series, Eppendorf, Madrid, Spain) for 24 h. Consequently, neutral red (150 µL) was added, and wells were incubated at 37 ℃ for 2 h, then, cells were washed, and plates were shaken with a destaining solution containing (1% acetic acid; 49% deionized water; 50% ethanol; 50 µL/well). Absorbance was determined at 540 nm in a UV-Vis spectrophotometer (PG Instrument Ltd. UK) [55]. The inhibition (%) was assessed by the following Equation (2):

The inhibition (%) = 100 − (O.D _Control_ − O.D _Treatment_)/(O.D _Control_) (2)

where: O.D = Optical density; IC50 values were calculated online (www.aatbio.com/tools/ic50-calculator), (Accessed on 25 July 2022).

### 4.8. Bentonite Characterization

#### 4.8.1. X-ray Diffraction (XRD) Analysis

X-ray powder diffractometry was performed by (Schimadzu 7000 diffractometer, Kyoto, Japan) to determine the structure of the four several types of bentonites (HAFR 1, 2, 3 and 4).

#### 4.8.2. Fourier-Transform Infrared (FTIR)

The FTIR apparatus (Bruker Tensor II FT-IR Spectrometer) was utilized with a resolution (cm^−1^) with a scan range of 4000–650 cm^−1^. The resulting spectrum represents molecular absorption, which generates a molecular fingerprint of the four various bentonite types (HAFR 1, 2, 3 and 4). The FTIR approach was conducted by mixing ethanol drops with some particles of bentonites on a glass slide, then placing it on a heater plate at 150 °C for 1 min to obtain a thin layer surface. The ready samples were kept in cold places and dark conditions before the analysis.

#### 4.8.3. Scanning Electron Microscopy (SEM)

The SEM is employed to scan a finely focused electron beam across the surface of a specimen. Specimens were magnified to 300,000×, while maintaining a large depth of focus. The ease of sample scanning by SEM (JEOL JSM 6360LA, Tokyo, Japan) over large distances is quite appealing, in that a large sample viewing area is first surveyed at generally low magnification to seek out particular areas of interest, followed by the high magnification of those specific areas for subsequent detailed investigations.

#### 4.8.4. Transmission Electron Microscope (TEM)

High-resolution images were examined by TEM (a JEOL JEM 2100, Tokyo, Japan) operating at 200 kV. The particle size of the four various types of bentonites (HAFR 1, 2, 3 and 4) was acquired by examining TEM images via Image software (AutoTEM 5 Software) [56].

### 4.9. Data Analysis

The obtained data were evaluated by the SPSS statistical package, ver. 23 (IBM SPSS Statistics for Windows, IBM Corp., Armonk, NY, USA). The means ± standard deviation (SD) was utilized to express the data. A one-way analysis of variance (ANOVA) was employed applying the Duncan test for data analyses at a significant level (*p* < 0.01). One-way ANOVA test was used to compare the means of two or more independent groups to determine whether there was statistical evidence that the associated population means were significantly different.

## Figures and Tables

**Table 1:** Chemical composition of initial raw bentonites by transmission electron microscopy-energy dispersive X-ray spectroscopy.

	Element (%)							
	Na	Mg	Al	Si	Cl	K	Ca	Ti
HAFR (1)	0.30	1.92	8.70	45.13	0	0.77	0.91	0
HAFR (2)	3.81	0.93	13.86	35.17	0.45	0.43	0.37	0.28
HAFR (3)	0.63	1.67	12.18	26.99	0	0.65	0.50	0.37
HAFR (4)	0.08	0.10	0.45	0.95	0.05	0.05	0.05	0.07

**Table 2:** Concentration (ng/L) and frequency of detection of aflatoxins M_1_ in raw milk samples using HPLC.

	All Raw Samples				Contaminated Samples				
Species	N	Min–Max	Mean (±SD)	Median (Q1–Q3)	N (%)	N (%) > MRL	Min–Max	Mean (±SD)	Median (Q1–Q3)
Cow	50	nd–122.80	48.13 ± 34.58	51.05 (14.60–76.95)	44 (88)	26 (54)	3.70–122.80	54.69 ± 31.55	54.90 (25.47–79.80)
Camel	20	nd–142.40	70.0 ± 50.35	76.4 (9.82–115.37)	16 (80)	14 (70)	6.50–142.40	87.50 ± 39.73	82.59 (66.67–127.55)
Sheep	15	nd–320.3	145.93 ± 111.84	140.60 (26.40–230.70)	13 (86.66)	11 (73.33)	9.6–320.3	168.38 ± 102.46	160.10 (81.40–260.55)
Goat	15	nd–490.30	221.28 ± 153.11	234.50 (98.40–360.20)	14 (93.33)	13 (86.66)	5.60–490.30	237.08 ± 145.64	242.90 (118.05–367.67)
P1 = 0.001, P2 = 0.002									

N, Numbers of samples; Min-Max, minimum-maximum; ±SD, Standard deviation; Q1, 25th percentile; Q3, 75th percentile; nd, not detected; MRL, Maximum residue level as per the European Commission (EC) Regulation No 1881/2006 (EU); AFM_1_, 50 ng/L; nd, non-detectable. P1 = Probability values result from the nonparametric comparison among species (Kruskal–Wallis test) involving all samples. P2 = Probability values result from the comparison among frequencies (%) of detection (Fisher’s Exact test).

**Table 3:** Adsorption of AFM_1_ (100 ng/L) in PBS solution by various bentonite HAFR types (1, 2, 3 and 4).

Samples	0 h	0.5 h	1 h	2 h	3 h	6 h	12 h
PBS (−Ve)	0	0	0	0	0	0	0
PBS +(100 ng/L) AFM_1_ (+ve)	100	100	100	100	100	100	100
PBS + (1 g) bentonite (HAFR 1)	0	0	0	0	0	0	0
PBS + (1 g) bentonite (HAFR 2)	0	0	0	0	0	0	0
PBS +(1 g) bentonite (HAFR 3)	0	0	0	0	0	0	0
PBS + (1 g) bentonite (HAFR 4)	0	0	0	0	0	0	0
PBS + (100 ng/L) AFM_1_+ (0.5 g) bentonite (HAFR 1)	100	83	72	63	55	52	50
PBS +(100 ng/L) AFM_1_+ (1 g) bentonite (HAFR 1)	100	65	44	33	10	4	1.5
PBS + (100 ng/L) AFM_1_+ (0.5 g) bentonite (HAFR 2)	100	82	71	62	55	53	51
PBS + (100 ng/L) AFM_1_+ (1 g) bentonite (HAFR 2)	100	63	41	21	9	3	2
PBS + (100 ng/L) AFM_1_+ (0.5 g) bentonite (HAFR 3)	100	80	68	59	52	50	48
PBS + (100 ng/L) AFM_1_+ (1 g) bentonite (HAFR 3)	100	60	38	18	5	1	0
PBS + (100 ng/L) AFM_1_+ (0.5 g) bentonite (HAFR 4)	100	88	76	62	55	53	52
PBS + (100 ng/L) AFM_1_+ (1 g) bentonite (HAFR 4)	100	68	51	24	11	6	4

(−Ve), negative; (+Ve), positive; PBS, phosphate buffer saline.

**Table 4:** Adsorption percentage (%) of AFM_1_ (100 ng/L) in PBS solution by different bentonite HAFR types (1, 2, 3 and 4) at various time intervals.

Samples	0 h	0.5 h	1 h	2 h	3 h	6 h	12 h
	Adsorption (%)						
PBS (−Ve)	0	0	0	0	0	0	0
PBS +(100 ng/L) AFM_1_ (+ve)	0	0	0	0	0	0	0
PBS + (1 g) bentonite (HAFR 1)	0	0	0	0	0	0	0
PBS + (1 g) bentonite (HAFR 2)	0	0	0	0	0	0	0
PBS +(1 g) bentonite (HAFR 3)	0	0	0	0	0	0	0
PBS + (1 g) bentonite (HAFR 4)	0	0	0	0	0	0	0
PBS + (100 ng/L) AFM_1_+ (0.5 g) bentonite (HAFR 1)	0	17	28	37	45	48	50
PBS +(100 ng/L) AFM_1_+ (1 g) bentonite (HAFR 1)	0	35	56	77	90	96	98.5
PBS + (100 ng/L) AFM_1_+ (0.5 g) bentonite (HAFR 2)	0	18	29	38	45	47	49
PBS + (100 ng/L) AFM_1_+ (1 g) bentonite (HAFR 2)	0	37	59	79	91	97	98
PBS + (100 ng/L) AFM_1_+ (0.5 g) bentonite (HAFR 3)	0	20	32	41	48	50	52
PBS+ (100 ng/L) AFM_1_+ (1 g) bentonite (HAFR 3)	0	40	62	82	95	99	100
PBS + (100 ng/L) AFM_1_+ (0.5 g) bentonite (HAFR 4)	0	12	24	38	45	47	48
PBS + (100 ng/L) AFM_1_+ (1 g) bentonite (HAFR 4)	0	32	49	76	89	94	96

(−Ve), negative; (+Ve), positive; PBS, phosphate buffer saline.

**Table 5:** Adsorption of AFM_1_ (100 ng/L) in milk by bentonite HAFR (1 and 3) at various time intervals.

Samples	0 h	0.5 h	1 h	2 h	3 h	6 h	12 h
Milk (−Ve)	0	0	0	0	0	0	0
Milk +(100 ng/L) AFM_1_ (+ve)	100	100	100	100	100	100	100
Milk + (1 g) bentonite (HAFR 1)	0	0	0	0	0	0	0
Milk + (1 g) bentonite (HAFR 3)	0	0	0	0	0	0	0
Milk + (100 ng/L) AFM_1_+ (1 g) bentonite (HAFR 1)	100	67	46	35	13	7	5
Milk + (100 ng/L) AFM_1_+ (1 g) bentonite (HAFR 3)	100	63	40	20	7	5	1.5

(−Ve), negative; (+Ve), positive.

**Table 6:** Adsorption (%) of AFM_1_ (100 ng/L) in milk by bentonite HAFR (1 and 3).

Samples	0 h	0.5 h	1 h	2 h	3 h	6 h	12 h
	Adsorption (%)						
Milk (−Ve)	0	0	0	0	0	0	0
Milk +(100 ng/L) AFM_1_ (+ve)	0	0	0	0	0	0	0
Milk + (1 g) bentonite (HAFR 1)	0	0	0	0	0	0	0
Milk + (1 g) bentonite (HAFR 3)	0	0	0	0	0	0	0
Milk + (100 ng/L) AFM_1_+ (1 g) bentonite (HAFR 1)	0	33	54	65	87	93	95
Milk + (100 ng/L) AFM_1_ + (1 g) bentonite (HAFR 3)	0	37	60	80	93	95	98.5

(−Ve), negative; (+Ve), positive.

**Table 7:** Qualitative properties of the milk sample (g /100 mL).

Samples	Mean (SD)			
	Fat	Protein	Lactose	SNF
Milk (−Ve)	0.55 ^b^ ± 0.005	3.57 ^b^ ± 0.001	4.91 ^a^ ± 0.015	8.49 ^c^ ± 0.004
Milk +(100 ng/L) AFM_1_ (+ve)	0.56 ^b^ ± 0.011	3.56 ^b^ ± 0.004	4.81 ^b^ ± 0.011	8.51 ^b^ ± 0.00
Milk + (1 g) bentonite (HAFR 1)	0.56 ^b^ ± 0.004	3.58 ^b^ ± 0.003	4.72 ^c^ ± 0.012	8.56 ^a^ ± 0.003
Milk + (1 g) bentonite (HAFR 2)	0.55 ^b^ ± 0.012	3.54 ^c^ ± 0.002	3.84 ^f^ ± 0.005	8.48 ^c^ ± 0.001
Milk + (1 g) bentonite (HAFR 3)	0.58 ^a^ ± 0.005	3.59 ^a^ ± 0.003	4.75 ^c^ ± 0.015	8.52 ^b^ ± 0.002
Milk + (1 g) bentonite (HAFR 4)	0.59 ^a^ ± 0.001	3.61 ^a^ ± 0.005	4.86 ^ab^ ± 0.004	8.59 ^a^ ± 0.011
Milk + (100 ng/L) AFM_1_+ (1 g) bentonite (HAFR 1)	0.54 ^c^ ± 0.011	3.53 ^c^ ± 0.004	4.83 ^b^ ± 0.013	8.54 ^a^ ± 0.012
Milk + (100 ng/L) AFM_1_+ (1 g) bentonite (HAFR 2)	0.53 ^c^ ± 0.002	3.56 ^b^ ± 0.012	4.67 ^d^ ± 0.02	8.56 ^a^ ± 0.003
Milk + (100 ng/L) AFM_1_+ (1 g) bentonite (HAFR 3)	0.56 ^b^ ± 0.001	3.54 ^c^ ± 0.011	4.72 ^c^ ± 0.003	8.55 ^a^ ± 0.004
Milk + (100 ng/L) AFM_1_+ (1 g) bentonite (HAFR 4)	0.58 ^a^ ± 0.016	3.58 ^b^ ± 0.011	4.51 ^e^ ± 0.004	8.56 ^a^ ± 0.006

(−Ve), negative; (+Ve), positive; ±SD, standard deviation; SNF, solid non-fat; Values means carrying a different superscript small letter on the same column are significantly different (*p* < 0.01).

**Table 8:** Estimation of cytotoxicity of bentonite and IC_50_ (µg/mL).

Concentration (µg/mL)	HAFR (1)		HAFR (2)		HAFR (3)		HAFR (4)	
	Inhibition %	Viability %	Inhibition %	Viability %	Inhibition %	Viability %	Inhibition %	Viability %
500	100	0	100	0	100	0	100	0
250	85	15	98	2	99	1	98	2
125	72	28	92	8	97	3	91	9
62.5	62	38	88	12	94	6	87	13
31.25	44	56	73	27	86	14	77	23
15.6	29	71	56	44	79	21	71	29
7.8	16	84	41	59	66	34	58	42
3.9	8	92	36	64	51	49	52	48
1.95	6	94	21	79	42	58	43	57
0.97	2	98	10	90	6	94	34	66
IC_50_	57.1		16.5		6.92		11.35	

## Data Availability

The data presented in this study are available on request from the corresponding author.
